# The Problem of Rural Midwifery

**Published:** 1907-10-19

**Authors:** 


### Nursing Administration.

### THE PROBLEM OF RURAL MIDWIFERY.

Public attention is being roused at last to the grave condition of affairs confronting the rural population in the immediate future. Valuable statistics have been collected in more than one quarter, throwing much light on the circumstances under which women are attended in childbirth throughout the country, and it becomes increasingly evident that the provisions of the Midwives Act, which will come into force in 1910, are likely to cause in many localities acute complications not merely to the poor, but to medical men, and to Poor Law guardians. The position of affairs is often misunderstood, even by medical practitioners. The Act which came into force in 1905 practically divided all midwives into three classes. There were first those who were admitted to the Roll of Midwives by virtue of holding a recognised certificate. Secondly, there were those who were admitted to the Roll of Midwives without a certificate by virtue of having been at least a year in *bone fide* practice as midwife. And there were, thirdly, those who chose to go on exercising their calling intermittently without applying for admission to the Roll, and these, so long as they did not call themselves midwives, or give anyone the impression that they were certified to act as midwives, were left unmolested for the time being. It is these women who in 1910 are to be prevented from attending women in childbirth without medical aid, and inquiry has revealed how large a share they still take in the midwifery of sparsely populated districts. Such women do not attempt to live by the proceeds of their craft. They may deliver two or three, at most perhaps a dozen, women in the course of a year. Their skill, learned by rule of thumb, may, however, be well esteemed locally in the little village where they reside, and so far no substitute for these " wise women " has been found. At present in these scattered districts, on lonely moors, or the wide upland regions such as the Sussex or Berkshire Downs, such practising midwives, who yet are denied the title of midwife, continue to do a good deal of work. Many of them run very close to the law in the matter of " taking or using the name or title of midwife," but it is to nobody's interest to interfere so long as no flagrant irregularities take place. What will happen when in 1910 and after, every case attended by such women will render them liable to a fine of ten pounds? It is important to realise that midwifery in scattered districts never has provided and never can provide a living by itself for those who practise it. This fact has been rendered abundantly clear by the able Report recently issued by the Association for Promoting the Training and Supply of Midwives. The Report is the result of an " Enquiry into the Existing Supply and the Present Rate of Remuneration of Midwives in England and Wales." It seems that at least half the population are dependent on the skill of the midwife for their entry into the world. But what proportion of births are attended by women whose names are not on the Roll it is, of course, impossible to ascertain. The crux of the whole Report is the conclusion arrived at that the midwife can only be entirely self-supporting when " she can reach a population of 8,000 within four miles in every direction from her own home." She must be " a strong woman, who is a good cyclist and popular with her patients." " It will take her from five to ten years to work up such a practice, and during that time she will not be earning her livelihood, but will need assistance." This, of course, means that only near towns and in manufacturing districts will professional midwives be found when the laws of supply and demand are logically carried out. It also means that the district nurse subsidised by contributions from the philanthropist is generally provided where (from the purely midwifery point of view) she is least in request. It is the towns which are best able to support the district nurse. It is in the towns that the midwife is best able to get on unsubsidised. Among members of a very thin and widely scattered population it is hardly likely that means will be forthcoming to pay for the services of even a village nurse with the minimum of training requisite to qualify her for admission to the Roll of Midwives. What then will be the resort of the poorer mothers when the casual assistance which has hitherto been available for them in childbirth is discountenanced by the law? There will remain the medical officer of the district. Even at the low fee of a guinea this functionary is beyond the means of the labourer's wife raising a large family on some fifteen shillings a week. The fee cannot be paid, and if it could it would be a grave embarrassment to the doctor to be burdened with the midwifery of a district many miles in extent. We can see no other solution of the difficulty but the intervention of the Poor Law to rescue mothers from dire distress. It must be borne in mind that the only form in which relief is usually granted to women in labour is the offer of " coming in." The ease with which the London mother is provided for by competing institutions for the training of midwives, who regard her as material, may well be envied by the mother in country districts. For the latter the only alternative to engaging her own nurse or doctor is to undergo the humiliating experience of a workhouse confinement, and thus stamp her offspring for ever with the workhouse brand in a neighbourhood where nothing is ever forgotten. So intense is the feeling on this head that in most country unions the lying-in ward is rarely used except for the nomad class, and it is unheard-of for women of respectable antecedents, however destitute for the moment, to resort to Poor Law relief for this purpose. It would be a national calamity if this tone of feeling were changed. If the agricultural population is to be systematically pauperised, the heaviest of all blows to the independence of the most upright peasantry in the world will have been accomplished by the agency of a law designed for their protection.

(*To be continued.*)

